# Application of Teager–Kaiser Energy Operator in the Early Fault Diagnosis of Rolling Bearings

**DOI:** 10.3390/s22176673

**Published:** 2022-09-03

**Authors:** Xiangfu Shi, Zhen Zhang, Zhiling Xia, Binhua Li, Xin Gu, Tingna Shi

**Affiliations:** 1 College of Electrical Engineering, Zhejiang University, Hangzhou 310027, China; 2 Zhejiang Zheneng Lanxi Power Generation Co., Ltd., Jinhua 321199, China; 3 School of Electrical Engineering, Tiangong University, Tianjin 300387, China

**Keywords:** rolling bearing, early bearing fault diagnosis, Teager–Kaiser energy operator (TKEO), vibration analysis

## Abstract

Rolling bearings are key components that support the rotation of motor shafts, operating with a quite high failure rate among all the motor components. Early bearing fault diagnosis has great significance to the operation security of motors. The main contribution of this paper is to illustrate Gaussian white noise in bearing vibration signals seriously masks the weak fault characteristics in the diagnosis based on the Teager–Kaiser energy operator envelope, and to propose improved TKEO taking both accuracy and calculation speed into account. Improved TKEO can attenuate noise in consideration of computational efficiency while preserving information about the possible fault. The proposed method can be characterized as follows: a series of band-pass filters were set up to extract several component signals from the original vibration signals; then a denoised target signal including fault information was reconstructed by weighted summation of these component signals; finally, the Fourier spectrum of TKEO energy of the resulting target signal was used for bearing fault diagnosis. The improved TKEO was applied to a vibration signal dataset of run-to-failure rolling bearings and compared with two advanced diagnosis methods. The experimental results verify the effectiveness and superiority of the proposed method in early bearing fault detection.

## 1. Introduction

Rolling bearings are key components that support the rotation of motor shafts, performing an important role in almost all the types of rotating machinery. High speed, high load, and limited lubrication make rolling bearings the parts with a high failure rate among all the motor components. Therefore, bearing fault diagnosis, especially early fault diagnosis, performs great significance to the operation security of motors.

Many relevant analysis methods have been proposed in recent decades. Thermal analysis is a valuable approach for the non-intrusive detection. The thermal images are effective features to reflect the operation states of bearings and the many impressive efforts made [1,2,3]. Applications of the acoustic emission signals deserve attention as well [4,5,6]. As an irreplaceable advantage in particular conditions, the acoustic sensors are able to record the signals in distance from the monitoring objectives. This technology greatly depends on the characteristics of background noise, which limits its industrial applications sometimes. In a broader view, stator current signals and the stray flux signals have also been applied for bearing fault diagnosis [7,8,9,10], and even the feasibility of the diagnosis based on variable reluctance signals has been discussed [11].

Vibration analysis is one of the most widely used technologies for bearing condition monitoring and performs satisfactory results in the diagnosis accuracy. The dynamic characteristics of machinery can be richly expressed by vibration signals. So, the vibration analysis is an appropriate choice for the early fault diagnosis in this research. In a case of a rotating faulty bearing, with its rolling elements periodically passing over a defect, impulses with the corresponding bearing fault characteristic frequency are contained in its vibration signals. Hence time-domain statistical parameters which are sensitive to such impulses, such as kurtosis and amplitude of vibration signal, can act as common bearing fault characteristics. However, locations and causes of faults are not detectable by the methods only based on these time-domain statistical parameters [12]. Moreover, in the early stage of bearing faults, the weak impact caused by the defect is hardly reflected on time-domain statistical parameters.

Through comparison with the statistical parameters, the envelope analysis on bearing vibration signals has greater potential to detect more detailed information about weak fault characteristics. It treats bearing vibration signals as amplitude modulated (AM) signals. A commonly applied method called the Hilbert envelope analysis extracts the resonant band signal with strong impulses through a band-pass filter, and demodulates the filtered signal with Hilbert transform to construct an amplitude envelope signal. Finally, the fault characteristic frequencies are easily identified in the envelope spectrum. The major challenge for the Hilbert envelope analysis is to appropriately estimate the center frequency and the bandwidth of the filter. To improve its robustness and sensitivity, many researches combine Hilbert envelope analysis with various signal processing technologies such as spectral kurtosis (SK) [13,14], empirical mode decomposition (EMD) [15,16], and variational mode decomposition (VMD) [17], providing different schemes for setting optimal filters. Meanwhile, there are particular shortcomings in these technologies: SK is vulnerable on some strong impact industrial occasions; EMD has the problems of endpoint effect and mode mixing; and VMD is difficult to configure the optimal parameters. As a result, there occur further improvement schemes [18,19,20,21,22,23].

As an alternative solution to calculate the (squared) amplitude envelope, the Teager–Kaiser energy operator (TKEO) is proposed to demodulate the instantaneous amplitude and the instantaneous frequency from a mono-component amplitude-modulated and frequency-modulated (AM-FM) signal [24]. Especially for AM signals, including bearing vibration signals, the TKEO energy is approximate to the squared envelope. Therefore, the bearing fault characteristic frequencies can be identified in the Fourier spectrum of TKEO energy of bearing vibration signals when a defect exists. Combined with signal processing technologies, TKEO has demonstrated a great potential to improve the accuracy of bearing fault diagnosis. A. Galezia et al. separated the bearing vibration signal into multiple frequency bands and applied the demodulation based on TKEO, to isolate and trace the strongest modulation components in each band [25]. D.-H. Kwak et al. combined TKEO with minimum entropy deconvolution to enhance bearing fault characteristics [26]. M. Azergui et al. used statistical parameters to select the optimal frequency band for wavelet packet transform and use TKEO to detect the hidden impact [27]. In the research of X. Pei, et al., the vibration signals preprocessed with TKEO were used as the input of an unsupervised machine-learning model for the capture of richer fault diagnosis information [28]. Z. Wang, et al. developed two novel cyclostationary methodologies with high computational speed based on the effective demodulation by TKEO for mono-component modulated signals [29]. Some researches have noted the unique advantages of TKEO as well [30,31,32]: TKEO energy of the origin signal avoids the estimation of extra band-pass filters; calculation of TKEO energy only requires three adjacent samples, which is simple and computationally efficient; TKEO has high time resolution and hence is sensitive to the fault under high-speed; and TKEO considers both the amplitude and frequency modulation information thereby potentially leading to more reliable detection results. However, TKEO is susceptible to wideband noise, especially when the energy of noise is close to or even greater than that of the signal to demodulate, which probably reduces the accuracy of the early bearing fault diagnosis.

Based on the assumption that the bearing vibration signal is an AM signal, this paper focuses on the following work: (1) The influence of Gaussian white noise on TKEO envelope analysis is discussed through theoretical analysis and simulation experiments, providing a basis for the denoising processing in the bearing fault diagnosis based on TKEO analysis. (2) A denoising method suitable for bearing fault vibration analysis is proposed to reduce the white noise of original vibration signals and retain the fault information in the target signal. Then, improved TKEO, combined TKEO with the proposed denoising method, is introduced.

The paper hereafter is organized as follows: Section 2 introduces TKEO and analyzes the influence of the noise in bearing vibration signals on TKEO; Section 3 proposes a denoising method suitable for bearing vibration analysis, and accordingly provides the complete flow of improved TKEO; the experimental results of several early bearing fault cases are presented in Section 4. Section 5 concludes the paper.

## 2. TKEO Envelope Analysis

### 2.1. Envelope Analysis of the Vibration Signal

Motor bearing vibration signals can be considered as a joint action of two parts. One part is the vibration with specific physical significance related to the natural frequency or motor speed. The other part is ordinarily the noise generated by random vibration, measurement error, or other factors, which can be regarded as the white noise subject to Gaussian distribution. Then the motor vibration displacement signal can be modeled as:

(1) $$s\left(t\right)=s_{m}\left(t\right)+\eta_{\sigma }\left(t\right)$$

(2) $$\eta_{\sigma }\left(t\right)∼N\left(0,\sigma \right)$$

where $s_{m}\left(t\right)$ and $\eta_{\sigma }\left(t\right)$ present the two parts of the vibration displacement signal s(t), respectively. $\eta_{\sigma }\left(t\right)$ is Gaussian white noise with standard variance *σ*.

When a bearing fault occurs, the shaft rotation causes rollers passing through the defect point periodically to generate impulses with the fault characteristic frequencies. Each impulse appears as a very sharp rise and then decays with an approximately exponential envelope as its energy is dissipated by the internal damping. Namely, the impulse component, which is non-existent when the bearing is healthy, appears in $s_{m}\left(t\right)$ as a result of the joint action of natural vibration and fault impact. The impulse component is approximately expressed as:

(3) $$s_{i}\left(t\right)=a_{i}\left(t\right)\cos\left(2\pi f_{\mathrm{bf}}t\right)\cos\left(2\pi f_{b}t\right)=A_{i}\left(t\right)\cos\left(2\pi f_{b}t\right)$$

where $a_{i}\left(t\right)$ is the instantaneous amplitude of the impact component, $f_{\mathrm{bf}}$ is fault characteristic frequency, and $f_{b}$ is the natural vibration frequency of the bearing.

Generally, because $f_{b}\gg f_{\mathrm{bf}}$, the fault characteristic term $A_{i}\left(t\right)=a_{i}\left(t\right)\cos\left(2\pi f_{\mathrm{bf}}t\right)$ generated by roller impact can be regarded as a modulation signal, and the natural vibration frequency term $\cos\left(2\pi f_{b}t\right)$ can be regarded as a carrier signal. Thus, $s_{i}\left(t\right)$ is an AM signal. Therefore, modulated fault features or information can be extracted from bearing vibration signals by amplitude envelope analysis.

### 2.2. Teager–Kaiser Energy Operator

TKEO was proposed by Kaiser in 1990 to measure the energy of mechanical processes that generates a single time-variant signal. It is considered as a high-resolution energy estimation. The TKEO of a continuous-time signal s(t) is:

(4) $$\psi \left[s(t)\right]={\left[\dot{s}(t)\right]}^{2}-s\left(t\right)\ddot{s}\left(t\right)$$

where $\dot{s}=\frac{ds}{dt}$, $\ddot{s}=\frac{d\dot{s}}{dt}$.

For an AM-FM signal s(t)=A(t)cos[φ(t)] with a time-variant amplitude A(t) and a time-variant phase cos[φ(t)], its instantaneous frequency is $\omega \left(t\right)=\dot{\varphi }\left(t\right)$. TKEO is applied to s(t), i.e.,

(5) $$\psi \left[s(t)\right]={\left[A(t)\dot{\varphi }(t)\right]}^{2}+\cos^{2}\left[\varphi (t)\right]\left[{\dot{A}}^{2}\left(t\right)-A\left(t\right)\ddot{A}\left(t\right)\right]+\frac{1}{2}A^{2}\left(t\right)\ddot{\varphi }\left(t\right)\sin\left[2\varphi (t)\right]$$

The change in amplitude and frequency of the modulation signal is often much slower than that of the carrier signal, that is, $\dot{A}\left(t\right)\ll A\left(t\right)$, $\ddot{A}\left(t\right)\ll A\left(t\right)$, and $\dot{\omega }\left(t\right)=\ddot{\varphi }\left(t\right)\ll \omega \left(t\right)$. So in Equation (5), the terms of $\cos^{2}\left[\varphi (t)\right]\left[{\dot{A}}^{2}\left(t\right)-A\left(t\right)\ddot{A}\left(t\right)\right]$ and $\frac{1}{2}A^{2}\left(t\right)\ddot{\varphi }\left(t\right)\sin\left[2\varphi (t)\right]$ can be omitted. Then Equation (5) is simplified as:

(6) $$\psi \left[s(t)\right]\approx A^{2}\left(t\right)\omega^{2}\left(t\right)$$

That means, TKEO extracts information about the modulation signal from the AM-FM signal by tracking the product of instantaneous amplitude and instantaneous frequency. For the single AM signal, its TKEO energy ψ[s(t)] is approximate to the squared envelope. Hence TKEO becomes a tool to demodulate fault characteristic signals as envelope analysis in bearing fault diagnosis.

In engineering practice, it is accelerometers that are the measurement tools of vibration signals. The measured (acceleration) signals x(t) and the bearing vibration displacement signal s(t) subject to $x\left(t\right)=\ddot{s}\left(t\right)$. According to s(t)=A(t)cos[φ(t)], it yields to:

(7) $$x\left(t\right)\approx -A\left(t\right)\omega^{2}\left(t\right)\cos\left[\varphi (t)\right]=-\omega^{2}\left(t\right)s\left(t\right)$$

If TKEO is applied to x(t) and the similar approximation is made above, then we have:

(8) $$\psi \left[x(t)\right]=\psi \left[\ddot{s}(t)\right]\approx A^{2}\left(t\right)\omega^{6}\left(t\right)$$

That is, when TKEO is used to demodulate an acceleration signal, TKEO energy, or signal energy defined by TKEO, is the product of the instantaneous amplitude of the vibration displacement signal to power 2 and the corresponding instantaneous frequency to power 6. According to Equation (3), the instantaneous amplitude A(t) contains the impulses with the fault characteristic frequency $f_{\mathrm{bf}}$. Therefore, $f_{\mathrm{bf}}$ and its higher harmonics in TKEO energy is a feasible indicator of the bearing fault.

For a discrete acceleration signal, the discrete version of the TKEO is provided in [24] by:

(9) $$\psi \left[x(n)\right]={\left[x(n)\right]}^{2}-x\left(n-1\right)x\left(n+1\right)$$

### 2.3. Influence of Noise on TKEO

Although TKEO shows unique advantages in bearing fault diagnosis, Gaussian white noise easily interferes with the bearing fault detection by TKEO energy. The analysis is as follows:

As shown in Equation (1), bearing vibration signals can be considered as two parts acting together. The first contains effective information about possible faults, the energy of which is often focused near a series of specific frequencies with physical meaning. Moreover, the second is the Gaussian white noise.

Let the ideal vibration accelerate signal $x_{\mathrm{id}}$ be corrupted by additive zero-mean Gaussian white noise $\eta_{\sigma }$ of standard deviation σ. The signal with noise is then provided by $x=x_{\mathrm{id}}+\eta_{\sigma }$, and ψ[x(n)] is the TKEO energy of x at the sample point n. Then after simplification, the expectation and the variance of ψ[x(n)] are subject to

(10) $$E\psi \left[x\left(n\right)\right]=E\psi \left[x_{\mathrm{id}}\left(n\right)\right]+\sigma^{2}$$

(11) $$\begin{array}{ll} D\psi \left[x\left(n\right)\right]= & D\psi \left[x_{\mathrm{id}}\left(n\right)\right]+\sigma^{2}Dx_{\mathrm{id}}\left(n-1\right)+\sigma^{2}Dx_{\mathrm{id}}\left(n+1\right)+\sigma^{2}{\left[Ex_{\mathrm{id}}\left(n-1\right)\right]}^{2}+\sigma^{2}{\left[Ex_{\mathrm{id}}\left(n+1\right)\right]}^{2}+4\sigma^{2}Dx_{\mathrm{id}}\left(n\right)\\ & +4\sigma^{2}{\left[Ex_{\mathrm{id}}\left(n\right)\right]}^{2}+3\sigma^{4} \end{array}$$

where E is the expectation operator and D is the variance operator.

$x_{\mathrm{id}}\left(n-1\right)$, $x_{\mathrm{id}}\left(n\right)$, $x_{\mathrm{id}}\left(n+1\right)$ are just different expressions of the same signal, so let $Dx_{\mathrm{id}}\left(n\right)=\sigma_{x}^{2}$, and then statistically $Dx_{\mathrm{id}}\left(n+1\right)=Dx_{\mathrm{id}}\left(n-1\right)=\sigma_{x}^{2}$ as well. Because $x_{\mathrm{id}}$ is a vibration acceleration signal, $Ex_{\mathrm{id}}=0$, and $Ex_{\mathrm{id}}\left(n+1\right)=Ex_{\mathrm{id}}\left(n-1\right)=0$. Then Equation (11) is simplified as

(12) $$D\psi \left[x\left(n\right)\right]=D\psi \left[x_{\mathrm{id}}\left(n\right)\right]+6\sigma_{x}^{2}\sigma^{2}+3\sigma^{4}$$

Thus the expectation and the variance of TKEO energy are seriously distorted by $\eta_{\sigma }$, especially when the variance $\sigma^{2}$ of $\eta_{\sigma }$ is close to or even above $Dx_{\mathrm{id}}=\sigma_{x}^{2}$, which is unfortunately a common situation in early bearing fault diagnosis. The impact caused by the early bearing fault usually carries very little energy and the signal-to-noise ratio of fault features in vibration signals is quite low for the fault diagnosis only with the identification of characteristic frequency $f_{\mathrm{bf}}$ in the TKEO energy spectrum.

From another perspective, $f_{\mathrm{bf}}$ is commonly far less than the sampling frequency and the natural bearing vibration frequency, so fault information in TKEO energy ψ[x] is mostly in the lower frequency band ($<10f_{\mathrm{bf}}$). According to Equation (6), the square envelope of instantaneous amplitude, $A^{2}\left(t\right)$, receives a gain by the 6th power of the instantaneous frequency, $\omega^{6}\left(t\right)$, after TKEO. Compared with high-frequency noise, medium and low-frequency modulated signals are far less gained, which reduces the reliability of diagnosis.

Some factors such as cogging effects and torque ripples also form peaks with specific frequencies in Fourier spectrum. Different from white noise, these peaks generally interfere little with bearing fault diagnosis and identification because their frequencies are inconsistent with the bearing fault characteristic frequencies. Therefore, Gaussian white noise in original vibration signals has most serious implications for TKEO analysis. Removal of white noise before TKEO can greatly improve the accuracy and sensitivity of early fault diagnosis.

### 2.4. Simulation Validation

This section validates the analysis in Section 2.3 using simulation signals with different content of white noise. The ideal vibration acceleration signal without Gaussian noise under bearing fault can be modeled as follows [33]:

(13) $$x_{\mathrm{id}}\left(t\right)=0.2\sin\left(2\pi f_{1}t\right)+0.1\sin\left[2\pi \left(2f_{1}\right)t\right]+\sum_{i=1}^{3}a_{i}e^{-\tau_{i}t^{\prime }}\sin\left(2\pi f_{\mathrm{bi}}t\right)$$

(14) $$t^{\prime }=\mathrm{mod}\left(t,\frac{1}{f_{\mathrm{bf}}}\right)$$

where $f_{1}$ is the rotating frequency of the shaft. $f_{\mathrm{bi}}$, i=1,2,3 are three different frequency resonances of the bearing. $\alpha_{i}$ and $\tau_{i}$ are corresponding resonance amplitudes and attenuation factors, respectively. $t^{\prime }=\mathrm{mod}\left(t,\frac{1}{f_{\mathrm{bf}}}\right)$ is the remainder of time variable t divided by $\frac{1}{f_{\mathrm{bf}}}$.

**Figure 1:**
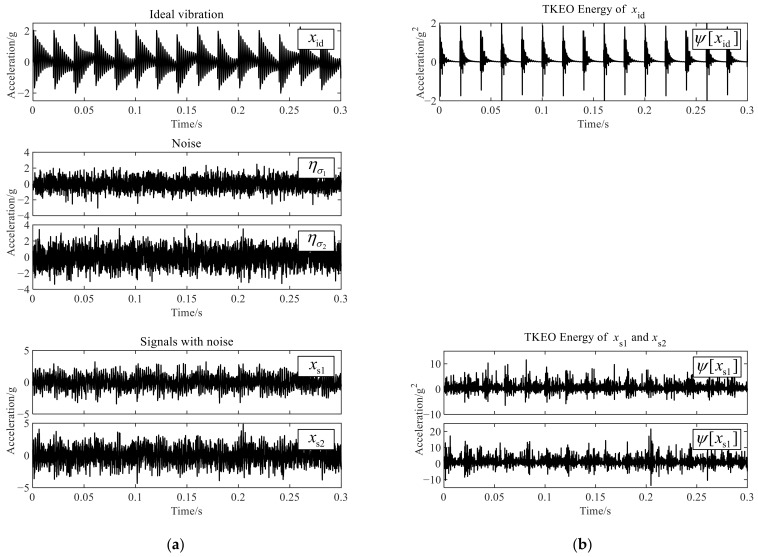
Waveforms of simulation signals and TKEO energy. (**a**) The ideal vibration signal, additive Gaussian noise and the signals with noise; (**b**) the TKEO energy of the ideal vibration signal and the signals with noise.

The parameter settings of simulation signals are shown in Table 1. The unit of signals is set to the gravity acceleration, g, consistent with common accelerometers. Sampling frequency $f_{s}=10\mathrm{kHz}$. $\sigma_{x}$ is the standard deviation of $x_{\mathrm{id}}$. $\eta_{\sigma_{1}}$ and $\eta_{\sigma_{2}}a$ are Gaussian white noise with the standard deviation of $\sigma_{1}$ and $\sigma_{2}$, respectively. The signals with noise, $x_{s1}$ and $x_{s2}$, are generated by adding $\eta_{\sigma_{1}}$ or $\eta_{\sigma_{2}}$ to $x_{\mathrm{id}}$, respectively, which are used to compare the impact of Gaussian white noise on TKEO. $x_{\mathrm{id}}$ is subtracted from its average value so that its expectation is 0. All simulation signals above are shown in Figure 1a and the TKEO energy of $x_{\mathrm{id}}$, $x_{s1}$, and $x_{s2}$, provided by Equation (9), is shown in Figure 1b.

**Figure 2:**
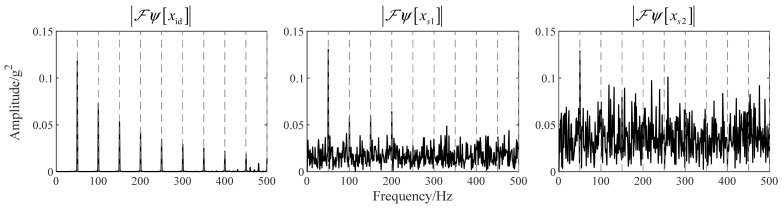
The TKEO energy spectra of $x_{\mathrm{id}}$, $x_{s1}$ and $x_{s2}$.

As shown in Table 2, the theoretical results calculated by Equations (10) and (12) are compared with the simulation results calculated from Figure 1. There are slight errors which can be ignored between the theoretical and simulation results because of the randomness of noise. It indicates, for bearing vibration signals with severe Gaussian white noise, the expectation and variance of TKEO energy are seriously increased by dozens or even hundreds of times. The influence of noise is more intuitive in the amplitude spectra of TKEO energy. Let ℱ be the notation of Fourier transform, and then the TKEO energy spectra, i.e., $\left|ℱ\psi \left[x_{\mathrm{id}}\right]\right|$, $\left|ℱ\psi \left[x_{s1}\right]\right|$ and $\left|ℱ\psi \left[x_{s2}\right]\right|$, are shown in Figure 2 (where the direct-current components are removed). The gray dashed lines represent the harmonic frequencies of $f_{\mathrm{bf}}$. The harmonics of $f_{\mathrm{bf}}$ are clearly displayed in $\left|ℱ\psi \left[x_{\mathrm{id}}\right]\right|$, which illustrates the demodulation function of TKEO. When the Gaussian noise with variance of $\sigma_{1}=\sigma_{x}$ is added into $x_{\mathrm{id}}$, its interferences pollute or mask the harmonics higher than the fourth order of $f_{\mathrm{bf}}$ in $\left|ℱ\psi \left[x_{s1}\right]\right|$. When the variance of the Gaussian noise comes to $\sigma_{2}=1.5\sigma_{x}$, only the fundamental frequency of $f_{\mathrm{bf}}$ is identified visually. In this way helpful information about the fault detection is covered up, and in other words, the TKEO analysis is seriously influenced by the Gaussian white noise in measured vibration signals.

## 3. Improved TKEO

### 3.1. Denoising Method Proposed for Vibration Signals

From the analysis in Section 2.3, to improve the accuracy of diagnosis by TKEO envelope and to detect bearing faults earlier possible, first the white noise should be removed (or suppressed) from vibration acceleration signals. According to Equation (1), to extract effective narrow-band fault information from original vibration signals, consider the following denoising problem about narrow-band signals:

(15) x(t)=u(t)+η(t)

where x(t) is original vibration signals measured by accelerometers. η(t) is additive zero-mean Gaussian noise. u(t) is the denoised target signal.

Equation (15) has the same form with a variational problem proposed in Reference [34]. Using L2 regularization to solve u, let the variational problem be

(16) $$\underset{x_{m}}{\min}\left\{{\left\|x-u\right\|}_{2}^{2}+\alpha {\left\|\frac{\partial u}{\partial t}\right\|}_{2}^{2}\right\}$$

where ${\left\|\cdot \right\|}_{2}$ is the notation of the L2 norm. α is penalty factor. Equation (16) is solved in Fourier domain, then:

(17) $$ℱ\left[u\left(t\right)\right]=\frac{ℱ\left[x\left(t\right)\right]}{1+\alpha \omega^{2}}$$

where Eu and Ex are approximately equal. ℱ is the notation of Fourier transform.

Equation (17) is regarded as a signal filter to x, and u as the filtering result. Then the filter transfer function H in the Fourier domain is

(18) $$H\left(\omega ,\omega_{c}\right)=\frac{1}{1+\alpha {\left(\omega -\omega_{c}\right)}^{2}}$$

where $\omega_{c}$ presents the center frequency. α affects filter bandwidth. The bandwidth of H is $\left[\omega_{c}-\sqrt{\frac{\sqrt{2}-1}{\alpha }},\omega_{c}+\sqrt{\frac{\sqrt{2}-1}{\alpha }}\right]$. The filter H is to extract the narrow-band signal with the center frequency $\omega_{c}$ from a signal with Gaussian white noise.

#### 3.1.1. Extract Fault Characteristic Components

The filter H provided in Equation (18) separates narrow-band effective information from a signal with noise. When a discrete bearing acceleration signal is filtered by H, a narrow-band component signal including parts of effective information is extracted, with a residual signal left. If the residual signal continues to be filtered by H with different central frequencies iteratively, a series of component signals with different central frequencies are extracted. As the iteration number increases and components are extracted one by one, energy of the residual signal finally approaches 0. At this time, it is considered that all effective information in the original signal was extracted. Let $x_{0}\left(n\right)=x\left(n\right)$, and then the process above can be expressed as

(19) $$\left\{ \begin{array}{l} H_{k}\left(\omega \right)=\frac{1}{1+\alpha {\left(\omega -\omega_{k}\right)}^{2}}\\ ℱ\left[u_{k}\left(n\right)\right]=ℱ\left[x_{k-1}\left(n\right)\right]H_{k}\left(\omega \right)\\ x_{k}=x_{k-1}\left(n\right)-u_{k}\left(n\right)\\ \underset{k\to +\infty }{\lim}\frac{{\left\|x_{k}\left(n\right)\right\|}_{2}^{2}}{{\left\|x\left(n\right)\right\|}_{2}^{2}}=0 \end{array}\right.$$

where k presents a positive integer such as 1, 2, … and $x_{k}$ is the residual signal after k-th filtering. $u_{k}$ is the narrow-band component extracted from $x_{k-1}$ by the filter $H_{k}$. $\omega_{k}$ is the center frequency of $H_{k}$. α affects bandwidth of $H_{k}$.

When $\frac{{\left\|x_{k}\left(n\right)\right\|}_{2}^{2}}{{\left\|x\left(n\right)\right\|}_{2}^{2}}$ comes to 0, the original signal is completely decomposed into a series of components. If α is large enough, that is, the passband of the filter $H_{k}$ is narrow enough, the spectrum of each component hardly overlaps with each other, and thereby each component is considered to be approximately orthogonal. Due to the fact that the effective information in the original vibration signal occupies specific narrow frequency bands, it is extracted into some batches of component signals (i.e., $u_{k}$) and separated from white noise located in other frequency bands. However, the white noise is included in other batches of components. To denoise the original signal, it is necessary to screen out the components with more information about the bearing fault.

#### 3.1.2. Reconstruct the Target Signal

Through the method described in Equation (19), the original vibration signal x(t) is decomposed into a series of narrow-band components with different center frequencies. Further, a unified evaluation is expected to screen out the more valuable components with richer effective fault information and lower white noise content.

The energy of white noise is evenly distributed in the whole frequency domain while that of effective information is concentrated in several narrow bands. According to Equation (19), each component has the same bandwidth. If a component contains some effective information, then its energy, equal to the sum of effective information and white noise, is greater than the energy of components with only white noise. Therefore, it is considered that for each component signal, its energy is negatively correlated with its proportion of white noise. With relatively large energy, the component is likely to contain more effective information and less white noise.

For real bearing vibration signals, some extracted components are certainly hard to trace or explain, or are even not related to the fault, while they are with large energy concentrated on a narrow band, similarly to the fault information. Obviously, these components are very likely caused by the vibration with specific physical significance such as converter noise, cogging effects and so on because white noise does not tend to forming peaks in the spectrum. A feasible attempt is to regard these components as a kind of “fault information” as well even if the reason for these components is unknown, because components with specific frequencies influences little on TKEO analysis, which is mentioned in the end of Section 2.3.

The Pearson correlation coefficient is an index to measure the linear correlation degree between two signals, which can be used to screen effective components. The correlation coefficient $\rho_{k}$ is defined between the component $u_{k}$ and the original vibration signal x as

(20) $$\rho_{k}=\rho \left(u_{k},x\right)=\frac{\langle u_{k}-Eu_{k},x-Ex\rangle }{\sqrt{{\left\|u_{k}-Eu_{k}\right\|}_{2}^{2}{\left\|x-Ex\right\|}_{2}^{2}}}$$

where 〈,〉 represents the inner product of two signals. $Eu_{k}$ and Ex are the expectations of $u_{k}$ and x, respectively. $\rho_{k}\in \left[-1,1\right]$, $\rho_{k}>0$ indicates a linear positive correlation of two signals, and $\rho_{k}<0$ indicates a linear negative correlation. $\rho_{k}=0$ indicates that the two signals are orthogonal.

Since the vibration acceleration signal x is satisfied to Ex=0, there is $Eu_{k}\approx 0$. Then Equation (20) is approximately seen as

(21) $$\rho_{k}\approx \frac{\langle u_{k},x\rangle }{\sqrt{{\left\|u_{k}\right\|}_{2}^{2}{\left\|x\right\|}_{2}^{2}}}=\frac{\langle u_{k},x\rangle }{{\left\|u_{k}\right\|}_{2}{\left\|x\right\|}_{2}}$$

Moreover, when α is large enough, ${\left\{u_{k}\left(n\right)\right\}}_{k=1,2,\cdots }$ are approximate orthogonal with each other, i.e.,

(22) $$\langle u_{i},u_{j}\rangle \approx 0,\;i\neq j$$

The final residual can be omitted because of $\underset{k\to +\infty }{\lim}\frac{{\left\|x_{k}\left(n\right)\right\|}_{2}^{2}}{{\left\|x\left(n\right)\right\|}_{2}^{2}}=0$. Then we have

(23) $$\rho_{k}\approx \frac{\langle u_{k},\sum_{m}u_{m}\rangle }{{\left\|u_{k}\right\|}_{2}{\left\|x\right\|}_{2}}=\frac{\sum_{m}\langle u_{k},u_{m}\rangle }{{\left\|u_{k}\right\|}_{2}{\left\|x\right\|}_{2}}\approx \frac{\langle u_{k},u_{k}\rangle }{{\left\|u_{k}\right\|}_{2}{\left\|x\right\|}_{2}}=\frac{{\left\|u_{k}\right\|}_{2}^{2}}{{\left\|u_{k}\right\|}_{2}{\left\|x\right\|}_{2}}=\frac{{\left\|u_{k}\right\|}_{2}}{{\left\|x\right\|}_{2}}$$

It is considered that $\rho_{k}$ has a positive correlation with the energy of $u_{k}$ and hence a negative correlation with the content of white noise, so $\rho_{k}$ works for screening the component signals with low white noise.

If each $\rho_{k}$ is assigned as a weight to relative $u_{k}$, the denoised target signal is reconstructed by

(24) $$\hat{x}\left(n\right)=\sum_{k}\rho_{k}u_{k}\left(n\right)$$

of which the components with specific frequencies or physical meaning are retained and white noise that affects the demodulation of TKEO is greatly weakened. Then the total energy of the target signal and the original signal is normalized by

(25) $${\hat{x}}_{e}\left(n\right)=\hat{x}\left(n\right)\sqrt{\frac{{\left\|x\left(n\right)\right\|}_{2}^{2}}{{\left\|\hat{x}\left(n\right)\right\|}_{2}^{2}}}$$

In the TKEO energy of ${\hat{x}}_{e}$, white noise is suppressed and the weak fault characteristics hidden in the original vibration signal are highlighted, which is conducive to early bearing fault diagnosis.

### 3.2. Complete Flow of Improved TKEO Proposed

In Section 3.1, a denoising method suitable for bearing vibration analysis is proposed. Improved TKEO is the method combining TKEO analysis with this denoising method. It consists of the following three parts: extraction of fault characteristic components, reconstruction of the target signal and TKEO envelope analysis. Its complete process is provided as follows:

Part 1: Extraction of fault characteristic components.

Step 1 Input the signal x(n), and the parameter α>0, δ>0;
Step 2 Set $x_{0}\left(n\right)=x\left(n\right)$ and k=0;
Step 3 Calculate the discrete Fourier transform $ℱx_{k}\left(n\right)$;
Step 4 k=k+1;
Step 5 Obtain the center frequency $\omega_{k}$;
Step 6 $ℱ\left[u_{k}\left(n\right)\right]=\frac{ℱ\left[x_{k-1}\left(n\right)\right]}{1+\alpha {\left(\omega -\omega_{k}\right)}^{2}}$. Calculate the inverse discrete Fourier transform and obtain $u_{k}\left(n\right)$;
Step 7 $x_{k}\left(n\right)=x_{k-1}\left(n\right)-u_{k}\left(n\right)$;
Step 8 Judge whether $\frac{{\left\|x_{k}\left(n\right)\right\|}_{2}^{2}}{{\left\|x\left(n\right)\right\|}_{2}^{2}}<\delta$. If not, then go back to Step 3. If yes, stop the iterations and proceed to Part 2.

Part 2: Reconstruction of the target signal.

Step 9 Calculate $\rho_{k}$ for each $u_{k}$ by $\rho_{k}=\frac{\langle u_{k}-Eu_{k},x-Ex\rangle }{\sqrt{{\left\|u_{k}-Eu_{k}\right\|}_{2}^{2}{\left\|x-Ex\right\|}_{2}^{2}}}$;
Step 10 $\hat{x}\left(n\right)=\sum_{k}\rho_{k}u_{k}\left(n\right)$;
Step 11 ${\hat{x}}_{e}\left(n\right)=\hat{x}\left(n\right)\sqrt{\frac{{\left\|x\left(n\right)\right\|}_{2}^{2}}{{\left\|\hat{x}\left(n\right)\right\|}_{2}^{2}}}$;

Part 3: TKEO envelope analysis.

Step 12 Calculate the TKEO energy $\psi \left[{\hat{x}}_{e}\right]$;
Step 13 Calculate the TKEO spectrum $ℱ\psi \left[{\hat{x}}_{e}\right]$ if necessary for the fault identification.

**Figure 3:**
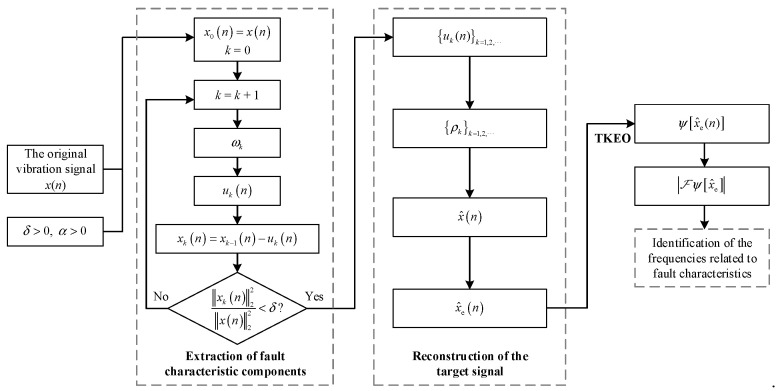
Flow chart of improved TKEO.

The flow chart is shown in Figure 3. Moreover, here is additional information for improved TKEO.

Part 1 is an iterative process, and its termination threshold is δ, the energy ratio of the residual signal to the original vibration signal. δ is commonly set within 0.01–0.2, depending on the noise level. In the filter $H_{k}$ of the k-th iteration, its center frequency $\omega_{k}$ is always set as the frequency corresponding to the maximum of $\left|ℱx_{k-1}\right|$, the magnitude Fourier spectrum of the current residual signal. It helps to extract all effective components with fewer iterations and calculation cost. The filter parameter α depends on signal sampling frequency and noise level. In practical application, if the calculation time of improved TKEO is necessarily further shortened, an additional termination condition K, the maximum number of extracted components, can be set to terminate the component extraction ahead.

In Part 2, there is no need to reconstruct the target signal after all the components are extracted. A recommended way is to merge the reconstruction into Part 1. Whenever a component signal $u_{k}$ is obtained in Step 6, its corresponding $\rho_{k}$ is calculated immediately and then $\rho_{k}u_{k}$ is added to reconstruct $\hat{x}\left(n\right)$. When the residual signal meets the terminal condition in Step 8, the finally obtained $\hat{x}\left(n\right)$ is equivalent to the result of Step 11. It avoids storing all the extracted components in the computer memory, and thereby the memory occupation is greatly reduced.

## 4. Experiment Data Analysis

### 4.1. Data Set

The data, acquired from the vibration signal dataset of run-to-failure rolling bearings published by Wang and Lei’s team of Xi’an Jiaotong University [35], were used to evaluate the feasibility and advantages of the proposed method for early bearing fault diagnosis. According to the experimental set shown in Reference [35], a hydraulic loading system was used to generate the radial force acting on the bearing seat of the test bearing. The greatly excessive vibration amplitude was used as the index of a thorough bearing failure. In each run-to-failure experiment, its motor operates ceaselessly until the maximum amplitude of the bearing vibration signal exceeded 10 times that of the initial healthy stage. The type of experimental bearings is LDK UER204. The vibration acceleration signal was sampled once every 1 min at the sampling frequency of 25.6 kHz, and the duration of each sampling was 1.28 s.

**Figure 4:**
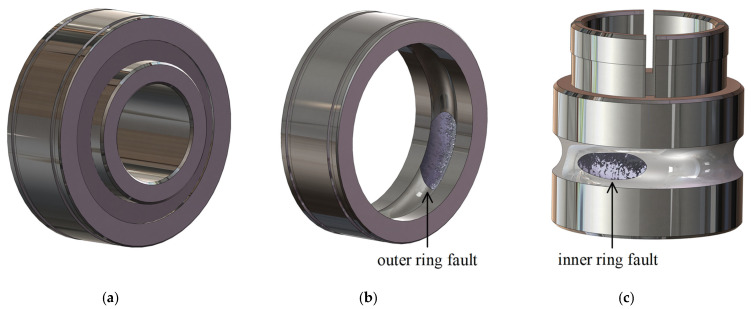
Sketch maps of the rolling bearings in typical stages. (**a**) In health; (**b**) with an outer ring fault; (**c**) with an inner ring fault.

The theoretical values of rolling bearing fault characteristic frequencies can be calculated by

(26) $$f_{\mathrm{bfo}}=\frac{1}{2}n_{b}f_{1}\left(1-\frac{d}{D_{m}}\cos\alpha_{b}\right)$$

(27) $$f_{\mathrm{bfi}}=\frac{1}{2}n_{b}f_{1}\left(1+\frac{d}{D_{m}}\cos\alpha_{b}\right)$$

where $f_{\mathrm{bfo}}$ is fault characteristic frequency of the outer ring. $f_{\mathrm{bfi}}$ is fault characteristic frequency of the inner ring. $f_{1}$ is the rotating frequency. $n_{b}$ is the number of rolling elements. d is the diameter of each rolling element. $D_{m}$ is the center diameter of the bearing cage. $\alpha_{b}$ is the contact angle. The sketch maps of the rolling bearings in typical stages are shown in Figure 4. The theoretical values of faulty bearing under various experimental conditions are presented in Table 3.

### 4.2. Outer Ring Defect

#### 4.2.1. Validation of Improved TKEO

The dataset mentioned in Section 4.1 provides the bearing vibration signals sampled every minute. Since the bearing fault is from scratch to serious, a task to find out when the initial fault exactly occurs is regarded as an appropriate validation with the support of these data. In this way, the feasibility of improved TKEO to detect bearing faults, especially early and weak faults, can be verified in detail.

**Figure 5:**
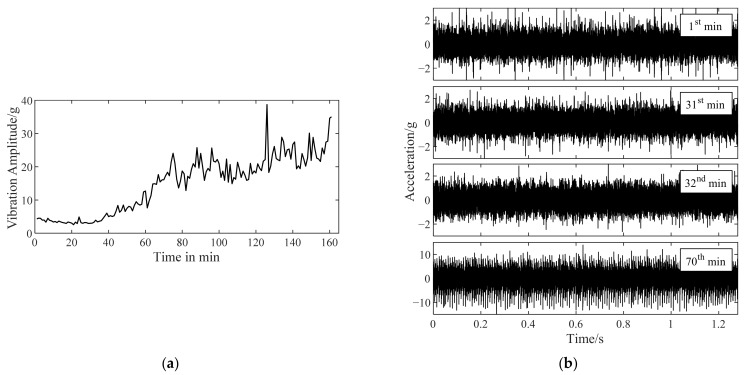
Vibration data of bearing 1 with a developing outer ring fault. (**a**) The curve of maximum vibration amplitude versus time; (**b**) vibration signals measured at 1st/31st/32nd/70th min.

The vibration data of bearing 1 in Table 3, from health to outer ring fault, are shown in Figure 5. The unit of vibration signal is the gravity acceleration, g (the same below). The maximum vibration amplitude in each sampling of bearing 1 is a fault characteristic to roughly estimate the bearing performance degradation. The curve of maximum vibration amplitude versus time is shown in Figure 5a. With the fault developing, an increasing trend of the maximum vibration amplitude emerges. It can be inferred that the bearing fault probably occurs near the 40th min and gradually deteriorate.

When a bearing generates vibration with an increased amplitude, it has operated with a latent fault for a while. It is reasonable to deduce that the initial fault occurs before the start point of the increasing vibration amplitude trend. Figure 5b shows the vibration signals of bearing 1 sampled at the beginning of the 1st, 31st, 32nd, and 70th min. In the following analysis, it can be seen that these signals correspond to the representative stage of health, pre fault, initial fault, and serious fault. More details about verifying when the initial fault occurs are recorded in Appendix A for purpose of avoiding a demonstrated cycle and increasing credibility.

**Figure 6:**
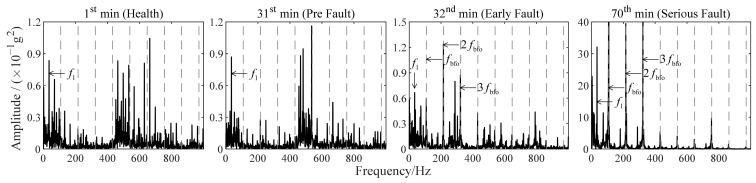
Fourier spectra obtained with improved TKEO for vibration signals in Figure 5b.

Improved TKEO is applied to the signals in Figure 5b, with its parameters set as α=0.05 and δ=0.1. Fourier spectra $\left|ℱ\psi \left[{\hat{x}}_{e}\right]\right|$ obtained with improved TKEO, i.e., TKEO energy spectra of the obtained target signal for each signal, are shown in Figure 6. The outer ring fault characteristic frequency $f_{\mathrm{bfo}}=107.9\mathrm{Hz}$. The gray dashed lines represent $f_{\mathrm{bfo}}$ or its higher harmonics $nf_{\mathrm{bfo}}$, where n is a positive integer.

It is found by the joint analysis of Figure 5b and Figure 6 that bearing 1 is in health at 1st min. At 31st min, the maximum vibration amplitude performs no significant increase, and the spectrum seems similar to that at 1st min, on which harmonics of $f_{\mathrm{bfo}}$ are not obviously reflected. However, the spectrum obtained from the vibration signal sampled at 32nd min changes a lot. There are distinct harmonics of $f_{\mathrm{bfo}}$ which are significantly different from the spectrum at 31st min. Combined with the following change in maximum vibration amplitude in Figure 5b, it can be concluded that an initial fault occurs in bearing 1 at 32nd min. With the gradual development of the fault, the maximum vibration amplitude further increases. By 70th min, bearing 1 is involved into a more serious fault. The harmonic components of $f_{\mathrm{bfo}}$ in $\psi \left[{\hat{x}}_{e}\right]$ are much greater than that of other frequencies now. Thus, the effectiveness and the feasibility of improved TKEO in the diagnosis of early outer ring fault are verified. The analysis results of improved TKEO align with the variations in maximum vibration amplitude, and additionally, can detect faults earlier than vibration amplitude monitoring.

#### 4.2.2. Parameter Setting of Improved TKEO

According to Section 3.2, the analysis result of improved TKEO mostly depends on the extraction of fault characteristic components. Two parameters require to be appropriately set in the component extraction part: α, which affects the bandwidth of bandpass filters; and δ, which mainly affects calculation time and the total number of components finally extracted.

In each iteration of extracting fault characteristic components, the center frequency $\omega_{k}$ of the filter $H_{k}$ is always selected as the frequency corresponding to the maximum magnitude of $\left|ℱx_{k-1}\right|$. It means that the components with physical meaning or fault information are more likely to be extracted first and hard to be remained in the residual signal at last. So, a larger δ tends to shorter calculation time and reduction in the total iterations, rather than a significant effect on the diagnosis accuracy of improved TKEO. The influence of α is different. If α decreases, the bandwidth of the filter $H_{k}$ becomes larger and thereby each extracted component signal carries additional noise within the passband. Worse, a too large bandwidth of $H_{k}$ probably results in a non-orthogonality of component signals. Thus, the reconstructed target signal retains more noise. In contrast, too large α increases the total component number because components with a smaller passband carry less energy, which causes excessive work. Consequently, the value of α is worth further discussion.

**Figure 7:**
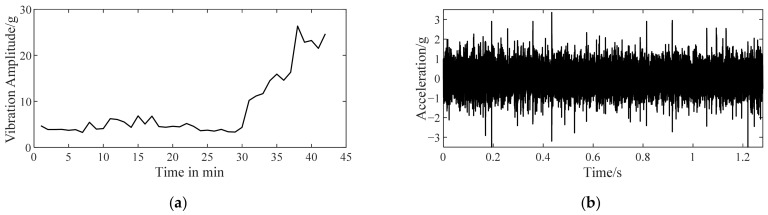
Vibration data of bearing 2. (**a**) The curve of maximum vibration amplitude versus time; (**b**) vibration signal measured at 9th min (initial fault).

**Figure 8:**
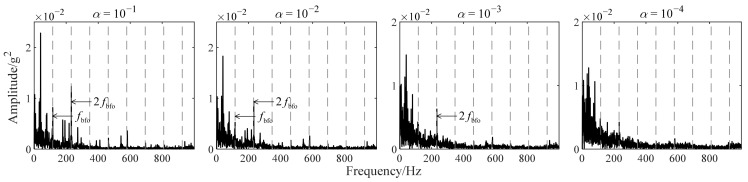
Fourier spectra obtained by improved TKEO with different α for the vibration signal in Figure 7b.

The vibration data (as shown in Figure 7) of bearing 2 in Table 3 were selected to describe the influence of α on improved TKEO in detail. An initial fault occurs in the outer ring of bearing 2 and is deduced with a similar method to Section 4.2.1. Figure 8 shows the spectra $\left|ℱ\psi \left[{\hat{x}}_{e}\right]\right|$ resulting from improved TKEO with different values of α. α is set to 10^−1^, 10^−2^, 10^−3^, and 10^−4^ while δ=0.1 invariably. The gray dashed lines represent $f_{\mathrm{bfo}}=115.6\mathrm{Hz}$, or its higher harmonics, $nf_{\mathrm{bfo}}$. It can be seen that as α decreases, $f_{\mathrm{bfo}}$ and its harmonic frequencies are gradually difficult to distinguish from the white noise in the spectrum $\left|ℱ\psi \left[{\hat{x}}_{e}\right]\right|$. Therefore, appropriately increasing the value of α probably improves the sensitivity of improved TKEO in early bearing fault diagnosis.

#### 4.2.3. Comparison with Existing Methods

In this section, three existing analysis methods for the bearing fault diagnosis are compared with improved TKEO: the method of singly using TKEO, the method of integrating empirical mode decomposition (EEMD) with spectral kurtosis (hereinafter referred to as EEMD-SK) [36], and the method of integrating genetic mutation particle swarm optimization (GMPSO) with VMD (hereinafter referred to as PSO-VMD) [37].

**Figure 9:**
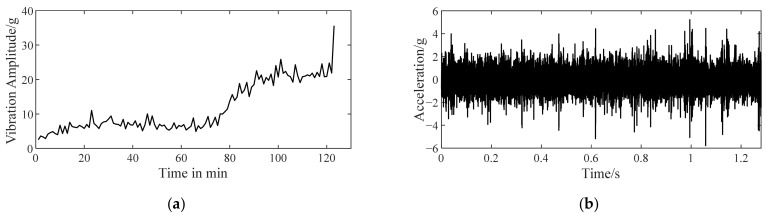
Vibration data of bearing 3. (**a**) The curve of maximum vibration amplitude versus time; (**b**) vibration signal measured at 66th min (initial fault).

**Figure 10:**
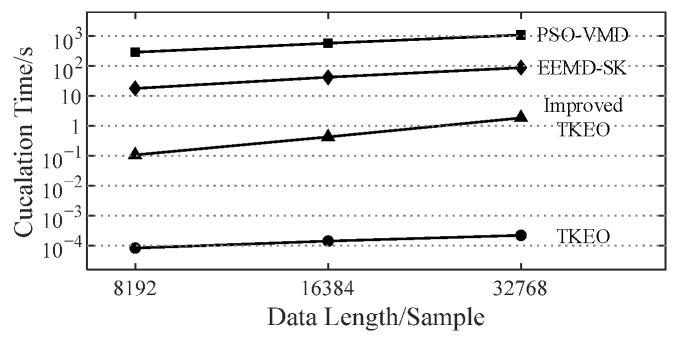
The average calculation time of mentioned methods (for the signal in Figure 9b).

**Figure 11:**
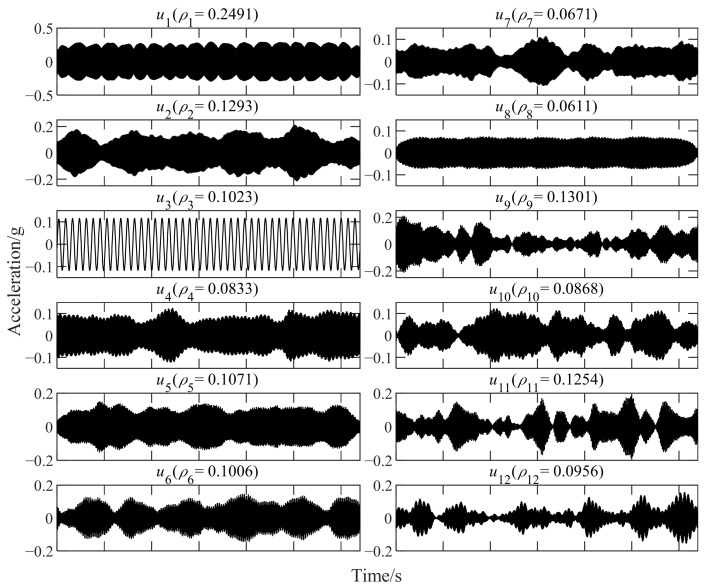
Some component signals extracted by improved TKEO (for the signal in Figure 9b).

**Figure 12:**
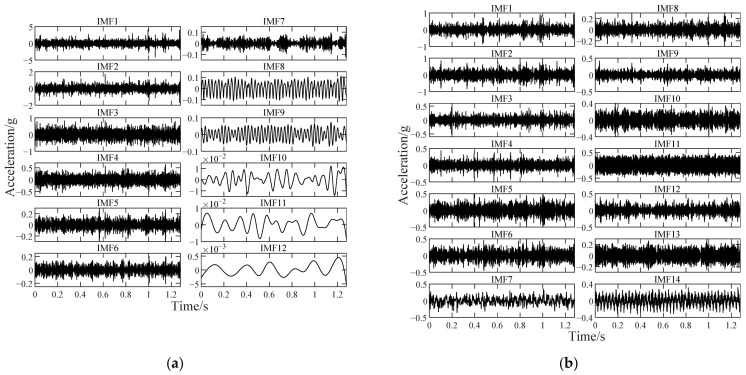
Decomposition results of EEMD and PSO-VMD (for the signal in Figure 9b). (**a**) Results of EEMD ($\frac{\sigma_{e}}{\sigma_{0}}=0.25$, 100 ensemble members); (**b**) results of VMD ($k_{v}=14$, $\alpha_{v}=63.1$).

**Figure 13:**
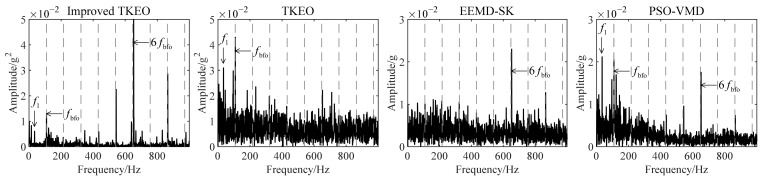
TKEO energy spectra or envelope spectra obtained with the four mentioned methods.

The vibration data (as shown in Figure 9) of bearing 3 with a developing outer ring fault for comparison are used as an example. The rotating frequency $f_{1}=35\mathrm{Hz}$ and the outer ring fault characteristic frequency $f_{\mathrm{bfo}}=107.9\mathrm{Hz}$. The main parameters of each method are set as Table 4. All calculations are completed by a desktop computer with i7-10700 (3.6 GHz). The average calculation time required for each method to analyze the signal in Figure 9b is shown in Figure 10. Some component signals extracted in the first part of improved TKEO are shown in Figure 11. The intrinsic modal functions (IMFs) as the decomposition results obtained by EEMD and PSO-VMD are shown in Figure 12. The TKEO energy spectra or envelope spectra for all the above methods are shown in Figure 13, where the gray dashed lines represent $f_{\mathrm{bfo}}$ or its higher harmonics $nf_{\mathrm{bfo}}$.

In Figure 13, there are obvious peaks located at the dashed lines in the energy spectrum obtained with improved TKEO, and the high-order harmonics of $f_{\mathrm{bfo}}$ are richly retained. The harmonics contribute to the identification of the early fault, and validate that the target signal reconstructed in improved TKEO reserves enough effective information for the fault diagnosis. For the same vibration signal, the spectrum obtained singly by TKEO only performs the fundamental frequency related to $f_{\mathrm{bfo}}$, and the higher harmonics therein are submerged, which provides less effective information for further diagnosis. From another view, one of the feathers of the Gaussian white noise is to form noise interferences at the bottom of the amplitude spectrum chart, and its average amplitude is related to its energy. The average amplitude of the noise at the bottom is much smaller in the Fourier spectrum obtained with improved TKEO. These act similarly to that in Section 2.4 and illustrate the denoising of improved TKEO.

In Figure 13, EEMD-SK and PSO-VMD extract some high-order frequency harmonics of $f_{\mathrm{bfo}}$, but their calculation time required is much longer than that of the improved TKEO, which affects the diagnosis efficiency. In EEMD, EMD is implemented many times on the original signal with different additional noise and the average is taken. Similarly, PSO-VMD is the process of applying VMD with different parameters to the original signal many times and finally using the best. Both methods carry out a lot of repeated calculations and thereby greatly increase the calculation time. However, in improved TKEO, during the part of extracting fault characteristic components, only one complete progress of iterations is expected, so the total calculation time is reduced.

### 4.3. Inner Ring Defect

**Figure 14:**
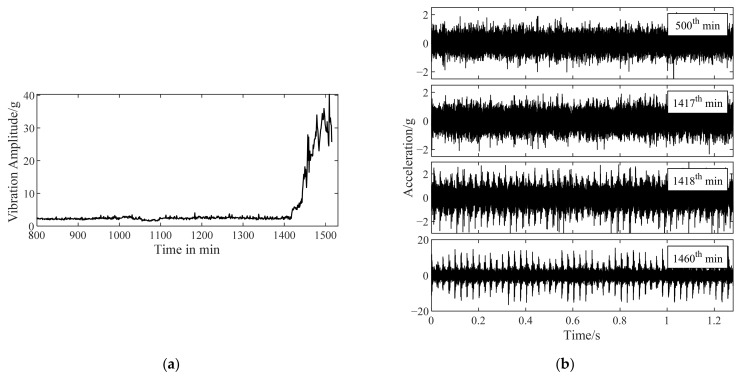
Vibration data of bearing 4 with a developing inner ring fault. (**a**) The curve of maximum vibration amplitude versus time; (**b**) vibration signals measured at 500th/1417th/1418th/1460th min.

The vibration data (as shown in Figure 14) of bearing 4 with a developing inner ring fault in Table 3 were selected to verify the feasibility of improved TKEO in inner ring fault diagnosis. The curve of maximum vibration amplitude versus time is shown in Figure 14a,b shows the vibration signals of bearing 1 sampled at the beginning of the 500th, 1417th, 1418th, and 1460th min. In the following analysis, it can be seen that these signals correspond to the representative stage of health, pre fault, initial fault, and serious fault, respectively.

**Figure 15:**
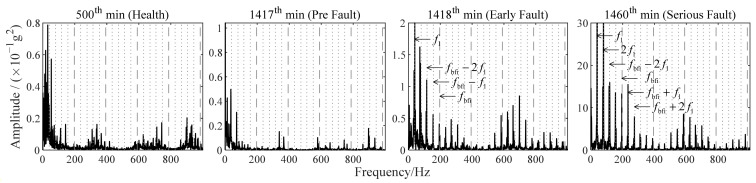
Fourier spectra obtained with improved TKEO for vibration signals in Figure 14b.

Improved TKEO was applied to the signals in Figure 14b, with its parameters set as α=0.05 and δ=0.1. Fourier spectra $\left|ℱ\psi \left[{\hat{x}}_{e}\right]\right|$ obtained with improved TKEO are shown in Figure 15. The rotating frequency $f_{1}=40\mathrm{Hz}$ and the inner ring fault characteristic frequency $f_{\mathrm{bfi}}=196.7\mathrm{Hz}$. The gray dashed lines represent $f_{\mathrm{bfi}}$ or its higher harmonics $f_{\mathrm{bfi}}$. The gray dotted lines present $nf_{\mathrm{bfi}}\pm f_{1}$ and $nf_{\mathrm{bfi}}\pm 2f_{1}$, which are the frequencies modulated by $nf_{\mathrm{bfi}}$ and $f_{1}$. Similar to the development of the outer ring fault, there is no frequency component related to $f_{\mathrm{bfi}}$ in the analysis result of the vibration signal sampled at 500th or 1417th min, indicating that the bearing is in health at this time. Then at 1418th min, the frequency components related to $f_{\mathrm{bfi}}$, not able to be identified before, appear in the spectrum of $\psi \left[{\hat{x}}_{e}\right]$. It is significantly different from the spectrum at 1417th min. With the joint analysis of Figure 14a, it can be concluded that the initial inner ring fault of bearing 4 occurs at the 1418th min. The fault develops over time and by 1460th min, frequency components relevant to $f_{\mathrm{bfi}}$ are more evident in the spectrum. Thus, it is verified that improved TKEO is also feasible to detect an inner ring fault in its early stage.

## 5. Conclusions

In this paper, the influence of Gaussian white noise on TKEO analysis was studied, and improved TKEO combined TKEO with a novel denoising method was proposed for the bearing fault vibration analysis. For the sampled bearing vibration signal, expectation and variance of the TKEO energy are extremely enlarged by the Gaussian noise therein. Consequently, a denoising processing is necessary for the bearing fault diagnosis based on TKEO analysis. Motivated by this opinion and the properties of bearing vibration signals, a novel denoising method was proposed, consisting of extracting fault characteristic components and reconstructing the target signal. Then, the improved TKEO method was put forward combined TKEO with the proposed denoising method. Improved TKEO was applied to explore when the initial fault occurred in a run-to-failure bearing vibration dataset and satisfactory results were obtained. The white noise was greatly reduced in the target signal. Moreover, for experiment bearings in fault, the harmonics of their fault characteristic frequencies were clearly identified in the Fourier spectrum obtained by improved TKEO. Further, discussions about the parameter settings indicate that appropriately increasing α enhances sensitivity to the early fault diagnosis in improved TKEO. The existing advanced methods of EEMD-SK and PSO-VMD were used for comparison experiments to confirm the considerable excellence of improved TKEO in calculation speed and accuracy. Therefore, the proposed improved TKEO method was verified to be feasible and convenient for the early bearing fault diagnosis.

At last, here are some discussions about what can be optimized in our research. First, the weights used for reconstructing the target signal were simply set to the Pearson correlation coefficients between the extracted components and the original signal. It is more a qualitative estimation of white noise content than an accurate deduction, which deserves further improvement. Second, this research is limited to exploring single point bearing faults. A compound bearing fault generates more unpredictable vibration signals and it becomes harder to detect the mutual modulation of fault characteristic frequencies. Further studies about compound failures are expected. Third, in our experimental validation of Section 4, whether the fault exists is identified visually by the amplitude spectrum. The visual identification method perhaps leads to the diagnosis results not accurate enough in some overly complicated cases. We will attempt to research on these problems in the future work.

## Figures and Tables

**Table 1:** Parameters of simulation signals.

Parameters					
$f_{b1}$ (Hz)	500	$f_{b2}$ (Hz)	2500	$f_{b3}$ (Hz)	4000
$\tau_{1}$ (s^−1^)	100	$\tau_{2}$ (s^−1^)	500	$\tau_{3}$ (s^−1^)	300
$a_{1}$ (g)	2	$a_{2}$ (g)	0.5	$a_{3}$ (g)	0.2
$f_{1}$ (Hz)	20	$f_{\mathrm{bf}}$ (Hz)	50	$f_{s}$ (Hz)	10,000
$\sigma_{x}$	0.7138	$\sigma_{1}/\sigma_{x}$	1	$\sigma_{2}/\sigma_{x}$	1.5

**Table 2:** The theoretical and simulation results of the expectation and variance of TKEO energy.

TKEO Energy	Simulation Results		Theoretical Results	
	Expectation	Variance	Expectation	Variance
$\psi \left[x_{\mathrm{id}}\right]$	0.1047	0.0836	/	/
$\psi \left[x_{s1}\right]$	0.6199	2.456	0.6142	2.420
$\psi \left[x_{s2}\right]$	1.244	7.468	1.251	7.531

**Table 3:** Theoretical values of fault characteristic frequencies for each bearing.

Number of Faulty Bearing	Radial Force (kN)	Motor Speed (r/min)	Fault Location	Fault Characteristic Frequency (Hz)
1	12	2100	Outer ring	107.9
2	11	2250	Outer ring	115.6
3	12	2100	Outer ring	107.9
4	10	2400	Inner ring	196.7

**Table 4:** Four mentioned methods and respective main parameter settings.

Analysis Methods	Parameter Settings			
Improved TKEO	Filter parameter α	0.05	/	
	Termination condition δ	0.1		
TKEO	/		/	
EEMD-SK	EEMD		Envelope Filter	
	$\frac{\sigma_{e}}{\sigma_{0}}$ ^1^	0.25	order	10
	Number of ensemble members	100		
PSO-VMD	GMPSO		VMD	
	Numbers of swarms	20	Termination condition	0.01
	Maximum generation	30		
	Parameters to optimize	$k_{v}$, $\alpha_{v}$^2^		

^1^$\frac{\sigma_{e}}{\sigma_{0}}$ presents the ratio of standard deviation of additional noise to that of the original signal in EEMD. ^2^ $k_{v}$ presents the number of modes decomposed in VMD. $\alpha_{v}$ presents the penalty factor in VMD.

**Figure A1:**
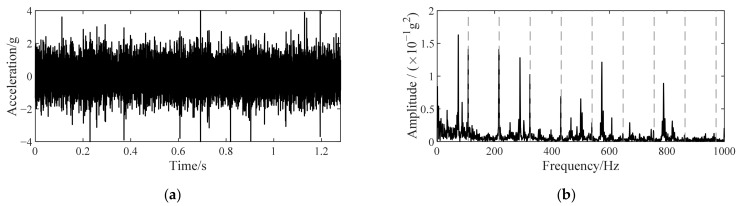
The signal sampled at 40th min and the spectrum obtained by improved TKEO. (**a**) The signal; (**b**) The spectrum obtained by improved TKEO.

**Figure A2:**
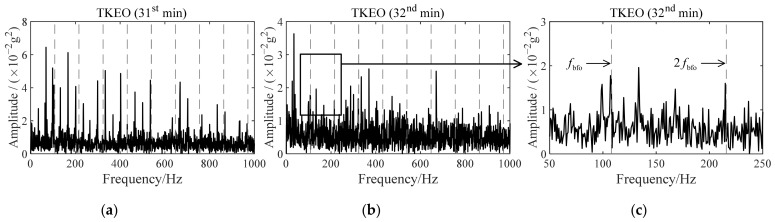
The spectrum obtained by TKEO. (**a**) For the signal sampled at 31st min; (**b**) for the signal sampled at 32nd min; (**c**) enlarged view of Figure A2.

**Figure A3:**
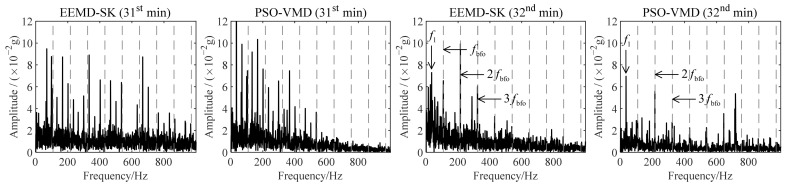
The spectrum obtained by EEMD-SK and PSO-VMD for the signals sampled at 31st and 32nd min.

## Data Availability

Not applicable.

## Nomenclature

AM	amplitude modulated
AM-FM	amplitude-modulated and frequency-modulated
D	the variance operator
E	the expectation operator
EEMD	ensemble empirical mode decomposition
EMD	empirical mode decomposition
ℱ	the notation of Fourier transform
GMPSO	genetic mutation particle swarm optimization
IMF	intrinsic modal function
SK	spectral kurtosis
TKEO	Teager–Kaiser energy operator
VMD	variational mode decomposition

## Appendix A

Section 4.2.1 is required to be verified more strictly when the initial fault occurs. Here is the detailed description about how to find the initial fault and the further validation by other analysis methods mentioned in Section 4.2.3.

First, credible examples for the fault and health stage of bearing 1 are necessary. According to Reference [35] which reported the dataset, bearing 1 is certainly in health at 1st min, and was damaged at the late stage of the sampling experiment. The credible examples are signals sampled at 1st and 70th min. Then the spectra obtained with improved TKEO are shown in Figure 6. At 1st min, most frequencies perform no relation with the fault characteristic frequency $f_{\mathrm{bfo}}=107.9\mathrm{Hz}$, while at 70th min, there are mainly the harmonics of $f_{\mathrm{bfo}}$ in the low frequency region as the evidence of the serious fault.

Second, it is noticed that an increasing trend of the maximum vibration amplitude begins near 40th min as mentioned in Section 4.2.1. Naturally, what to perform next is an analysis of the signal sampled at 40th min. The signal and the spectrum obtained by improved TKEO are shown in Figure A1, where the dashed lines represent the harmonic frequencies of $f_{\mathrm{bfo}}$. Similar to the spectrum at 70th min (shown in Figure 6), Figure A1b performs obvious harmonics of $f_{\mathrm{bfo}}$. It indicates a slighter fault because their amplitudes seem much smaller. So, the preliminary inference is that the initial fault occurs before 40th min.

Third, all the signals sampled before 40th min are checked one by one until there is no fundamental frequency of $f_{\mathrm{bfo}}$ in the spectrum. Then the spectra of the signal sampled at 31st and 32nd are noticed.

Fourth, the signals sampled at 31st and 32nd min are analyzed with other methods. The methods mentioned in Section 4.2.3 are chosen here, i.e., TKEO, EEMD-SK, and PSO-VMD. The results are shown in Figure A2 and Figure A3, where the dashed lines represent the harmonic frequencies of $f_{\mathrm{bfo}}$. In Figure A2, $f_{\mathrm{bfo}}$ firstly appears in the spectrum similarly to improved TKEO although it is hard to identify without an enlarged view. In Figure A3, although there are some frequency peaks seem to attach the dashed lines at 31st min, e.g., the peak near $3f_{\mathrm{bfo}}$, these frequencies do not perform their integer multiple harmonics, so the possibility that they are the fault characteristics is eliminated. That is why the harmonics and modulation sidebands are also important information or characteristics in the fault diagnosis. Then at 32nd min, the spectra obtained by both two methods reflect harmonics of $f_{\mathrm{bfo}}$, which is the main change in frequency components. The analysis results are consistent with improved TKEO. Thus, when the initial fault occurs, is verified.
